# First molecular characterization of *Cryptosporidium* and *Giardia* from bovines (*Bos taurus* and *Bubalus bubalis*) in Sri Lanka: unexpected absence of *C. parvum* from pre-weaned calves

**DOI:** 10.1186/1756-3305-7-75

**Published:** 2014-02-21

**Authors:** Harshanie Abeywardena, Aaron R Jex, Anson V Koehler, RPV Jayanthe Rajapakse, Kanchana Udayawarna, Shane R Haydon, Melita A Stevens, Robin B Gasser

**Affiliations:** 1 Faculty of Veterinary Science, The University of Melbourne, Parkville, Victoria, Australia; 2 Department of Veterinary Pathobiology, Faculty of Veterinary Medicine and Animal Science, University of Peradeniya, Peradeniya, Sri Lanka; 3 Melbourne Water Corporation, Victoria, Australia

**Keywords:** *Bos taurus*, *Bubalus bubalis*, *Cryptosporidium*, *Giardia*, Single-strand conformation polymorphism (SSCP) analysis, Restriction endonuclease fingerprinting (REF), Sri Lanka

## Abstract

**Background:**

The genetic characterization of *Cryptosporidium* and *Giardia* has important implications for investigating their epidemiology and underpins their control. We undertook the first molecular epidemiological survey of domestic bovids in selected regions of Sri Lanka to establish whether they excreted *Cryptosporidium* and/or *Giardia* with zoonotic potential.

**Methods:**

Faecal samples were collected from dairy calves (n = 340; *Bos taurus*; < 3 months of age; weekly sampling for six weeks) and water buffaloes (n = 297; *Bubalus bubalis*; <6 months and ≥6 months of age; one sampling) from seven different farms in Sri Lanka. Genomic DNAs were extracted from individual faecal samples and then tested for the presence of parasite DNA using a PCR-based mutation scanning-targeted sequencing-phylogenetic approach, employing genetic markers within the small subunit of nuclear ribosomal RNA and 60 kDa glycoprotein genes (designated p*SSU* and p*gp60*, respectively) for *Cryptosporidium*, and within the triose phosphate isomerise (p*tpi*) gene for *Giardia*.

**Results:**

Based on p*SSU* sequence data, *C. bovis*, *C. ryanae* and six new genotypes that were genetically similar but not identical to *C. andersoni* (n = 1), *C. bovis* (n = 1), *C. ryanae* (n = 3) and *C. suis* (n = 1) were recorded in cattle. For p*SSU*, two other, new genotypes were defined in water buffalo, which were genetically most similar to *Cryptosporidium* genotypes recorded previously in this host species in other countries including Australia. Consistent with the findings for p*SSU*, no species or genotypes of *Cryptosporidium* with zoonotic potential were detected using p*gp60.* Based on p*tpi* sequence data, *G. duodenalis* assemblages A and E were detected in four and 137 samples from cattle, respectively, and assemblage E in two samples from water buffaloes.

**Conclusions:**

The present study showed that *C. parvum*, the most commonly reported zoonotic species of *Cryptosporidium* recognised in bovine calves globally, was not detected in any of the samples from pre-weaned calves tested in the present study. However, eight new genotypes were recorded. Future studies of different host species in various regions are required to investigate the molecular epidemiology of cryptosporidiosis and giardiasis in Sri Lanka and neighbouring countries in South Asia.

## Background

*Cryptosporidium* and *Giardia* are two parasitic protists that mainly infect the gastrointestinal tract and cause enteric disease in humans and various other animals [1,2]. These protozoa are global in their distribution and adversely impact on human health in both developed and developing countries [3,4]. Infections are usually transmitted via the faecal-oral route, following direct or indirect contact with infective stages (oocysts or cysts) [2,3]. Although infections are often self-limiting in immuno-competent individuals [3,5], they can become severe and chronic in infants, elderly people or immuno-compromised or -suppressed individuals [6-8]. Key risk factors for cryptosporidiosis and giardiasis of humans include contact with individuals with diarrhoea, swimming in public pools, travel to developing countries and, importantly, direct contact with animals (e.g., [9-12]).

Cryptosporidiosis and giardiasis can be transmitted from human to human (anthroponotic) or from animal to human (zoonotic) [13]. Ruminants often have been implicated as a major source of human cryptosporidiosis and giardiasis based on the findings of many molecular epidemiological studies (reviewed in [14,15]). For instance, investigations of outbreaks and case–control studies [16-20] have demonstrated that there is a strong link between human cryptosporidiosis/giardiasis and the direct or indirect contact with cattle, particularly pre-weaned calves [15,21]. To date, seven species (i.e. *C. andersoni*, *C. bovis*, *C. felis*, *C. hominis*, *C. parvum*, *C. ryanae* and *C. suis*) and two genotypes of *Cryptosporidium* (i.e. “pig genotype II” and a new “*C. suis*-like genotype”) have been recorded in cattle [22,23]. For *Giardia duodenalis*, assemblage E is the most commonly reported genotype in cattle, followed by assemblages A and B [24-26]. An appraisal of the literature shows that the majority of molecular studies of *Cryptosporidium* and *Giardia* of cattle and other animals relates mainly to a limited number of countries in the developed world [3,27], but there is little published information for developing countries, including Sri Lanka. In addition, although there have been numerous studies of cattle (*Bos taurus*) in many developed regions of the world [27], there have been few investigations of the molecular epidemiology of cryptosporidiosis and giardiasis in related bovids, such as water buffaloes (*Bubalus bubalis*) [28,29], which are common domesticated animals in many countries, particularly in Asia.

Although *Cryptosporidium* and *Giardia* are known to present clinical problems in humans [30-32] and animals [33-35] in Sri Lanka, there is no detailed epidemiological information on the species and genotypes/assemblages of these parasites present in humans or any other animals. These knowledge gaps relate to the fact that suitable molecular methods have not yet been used previously to identify and characterise such species and genotypes/assemblages in this country. Therefore, in the present study, we conducted the first molecular epidemiological survey in Sri Lanka to establish whether domestic bovids might harbour *Cryptosporidium* and/or *Giardia* with zoonotic potential. We used a PCR-based approach employing genetic markers in the small subunit of ribosomal RNA (*SSU*), 60 kDa glycoprotein (*gp60*) genes for *Cryptosporidium* and in the triose phosphate isomerase (*tpi*) gene for *Giardia*, which have been widely used for the genetic characterization of members of these genera [3,27]; we were particularly focussed on identifying zoonotic genotypes of these protozoa.

## Methods

### Sample collection

Faecal samples were collected rectally from pre-weaned dairy calves (*Bos taurus*; < 3 months of age) and water buffaloes (*Bubalus bubalis*; <6 months and ≥6 months of age) from seven different farms (designated AB, NZ and DY; BR, HA, NK and MR) in Sri Lanka between August 2012 and February 2013. The three dairy cattle farms (AB, NZ and DY) studied were large, intensive farms, located in the highland wet zone (average >2000 mm of annual rainfall and > 900 m altitude), and maintained Ayrshire, Holstein Friesian and Jersey breeds, respectively. Faecal samples were collected weekly for six weeks from these three dairy farms. At the first visit, faeces were collected from 20 pre-weaned calves (1–12 weeks) from each farm, and individual calves were sampled weekly for 6 weeks, providing a total of 340 samples for molecular testing. In addition, a total of 297 faecal samples were collected once from the two different age groups of water buffalo. The four riverine buffalo herds (BR, HA, NK and MR) studied were in two different climatic zones, with an average of 1,750-2,500 and 1,850-5,000 mm of annual rainfall at altitudes of <300 and 300–900 m, respectively.

Cattle and buffaloes were born and raised on the farms studied, and fed whole milk (twice a day) until weaning at 3 months of age. Dairy calves were reared in individual pens, whereas buffalo calves were maintained as groups (n = 3–10) in pens. Therefore, there was no direct contact between calves and adult cattle or buffaloes. Herd management practices on these dairy and water buffalo farms are typical of large-scale, intensive farms in Sri Lanka.

### Isolation of genomic DNA from faecal samples, and PCR amplification

Genomic DNA was isolated from individual faecal samples using the PowerSoil DNA isolation kit (MoBIO, USA) [29], and then frozen at −20°C until molecular testing. Each genomic DNA sample was tested by PCR for *Cryptosporidium* DNA employing markers (designated p*SSU* and p*gp60*) within the small subunit nuclear ribosomal RNA and 60 kDa glycoprotein genes, and for *Giardia* DNA using a region (p*tpi*) within the triose phosphate isomerase gene [29,36]; p*gp60* is employed specifically for the detection and assignment of *Cryptosporidium* species, genotypes or subgenotypes that are infective to humans (cf. [2]). Some genomic DNAs (n = 50) were also tested for inhibition of the enzymatic reaction in PCR using a DNA-spiking approach [37]. In brief, aliquots (2 μl) of individual genomic DNA samples that had been test-negative in PCR were individually spiked with 1 pg of *C. parvum* DNA and subjected to PCR-based mutation scanning to demonstrate the amplification of a specific product. There was no evidence that samples tested contained constituents inhibitory to PCR.

### Mutation scanning-based sequencing and phylogenetic analyses of sequence data

For p*SSU* amplicons, single-strand conformation polymorphism (SSCP) analysis [38] was carried out as described previously [39]. For p*tpi* amplicons, restriction endonuclease fingerprinting was employed, using the enzyme *Rsa*I (Promega) [40-42]. Amplicons representing each banding profile were selected and treated with exonuclease I and shrimp alkaline phosphatase (Fermentas), according to the manufacturer’s instructions, and then sequenced in both directions by direct, automated sequencing (BigDye Terminator v.3.1 chemistry, Applied Biosystems, USA), using the same primers employed in secondary PCR. The quality of each sequence was assessed based on the corresponding electropherogram using the program BioEdit [43], and the sequences determined were compared with known reference sequences using the Basic Local Alignment Search Tool (BLAST; http://www.ncbi.nlm.nih.gov/BLAST). Phylogenetic analysis of sequence data was performed using the Bayesian inference (BI) tree building method in MrBayes 3.1.2 [44,45]. Posterior probabilities (pp) were calculated via 2,000,000 generations, utilizing four simultaneous tree-building chains, with every 100th tree being saved. At this point, the standard deviation of split frequencies was <0.01, and the potential scale reduction factor (PSRF) approached one. A 50% majority rule consensus tree for each analysis was constructed based on the final 75% of trees generated by BI.

Phylogenetic analysis was conducted to assess the relationships of the sequences from the present study to those available from public databases for species or genotypes of *Cryptosporidium*, and published in the peer-reviewed literature. In brief, species were determined based on 100% sequence homology to known species of *Cryptosporidium*. Phylogenetic analysis was used to provide unequivocal support for the classification of species and genotypes of *Cryptosporidium*; new genotypes were designated using sequential numbers, according to a previous study [29]. In addition, statistical analysis of proportional difference was performed using the Fisher’s exact test [46].

## Results

### *Cryptosporidium* species/genotypes in cattle

**Figure 1:**
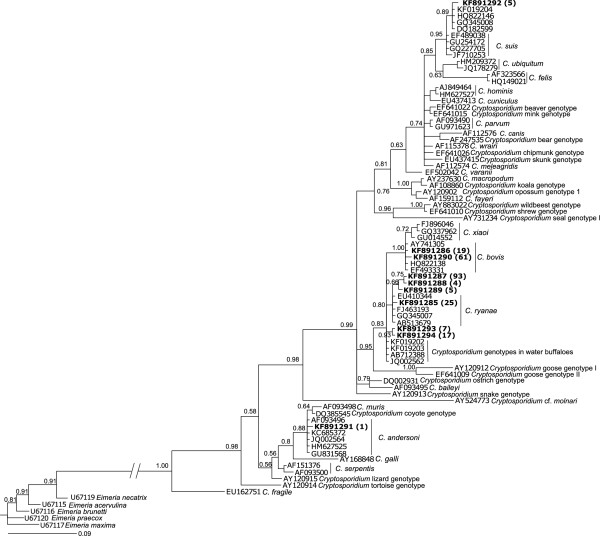
**Phylogenetic analysis of p*SSU *sequence data for *Cryptosporidium *using Bayesian inference (BI) analysis.** The ten distinct p*SSU* sequences determined in the present study and 36 reference sequences representing *Cryptosporidium* (accession nos. listed in Additional file 1) were included in the analysis. *Eimeria necatrix*, *E. acervulina*, *E. brunetti*, *E. praecox* and *E. maxima* sequences were used as outgroups. Accession numbers of publicly available sequences are indicated at the ends of the branches. Accession numbers linked to sequences determined in the present study are in bold-type; the numbers of samples with particular sequence types are in parentheses. Posterior probabilities (pp) for major nodes are indicated.

Although p*gp60* was not amplified from any of the 340 genomic DNA samples tested, the p*SSU* region was amplified from 211 (62.1%) of them. SSCP-based analysis of all amplicons revealed eight unique banding profiles. In total, 60 amplicons representing these eight profiles were sequenced (5–10 per profile). No sequence variation was detected among all sequences representing each SSCP profile, such that, ultimately, eight unique p*SSU* sequences (GenBank accession nos. KF891285-KF891292) were defined. These eight sequences differed by 72-99% upon pairwise comparison. Phylogenetic analysis (Figure 1) of these eight as well as 70 reference sequences (Additional file 1) included for comparative purposes revealed *C. bovis* (accession no. KF891286), *C. ryanae* (KF891285) and six new records (genotypes) that were genetically similar (72-99%) but not identical to *C. andersoni* (n = 1; accession no. KF891291), *C. bovis* (n = 1; KF891290), *C. ryanae* (n = 3; KF891287-KF891289) or *C. suis* (n = 1; KF891292), respectively (see Additional files 2 and 3). In the epidemiological context (Table 1), *C. bovis* and *C. ryanae* were detected in 5.6% and 7.4% of all samples only from farm AB, respectively, and the six new *Cryptosporidium* sequence types (accession nos. KF891287-KF891292) were detected in 0.3-27.6% of samples from farms AB, DY and/or NZ (Table 1).

**Table 1:** ***Cryptosporidium *** **species/genotypes and *Giardia duodenalis *** **assemblages detected in faecal samples from dairy cattle from three farms in Sri Lanka**

**Farm**	**Total**	***Cryptosporidium *** species or genotypes*								***Giardia duodenalis *** assemblages*	
		***C. ryanae *** **(KF891285)**	***C. bovis *** **(KF891286)**	**Genotype 4 (KF891287)**	**Genotype 5 (KF891290)**	**Genotype 6 (KF891288)**	**Genotype 7 (KF891289)**	**Genotype 8 (KF891291)**	**Genotype 9 (KF891292)**	**Assemblage A (KF891295)**	**Assemblage E (KF891296- KF891310)**
AB	117	25	19	22	9	1	0	0	0	0	60
NZ	120	0	0	31	38	0	0	0	2	0	39
DY	103	0	0	41	14	3	5	1	0	4	38
Total	340	25	19	94	61	4	5	1	2	4	137

*GenBank accession nos. are given in round parentheses.

### *Cryptosporidium* species/genotypes in water buffaloes

Although p*gp60* was not amplified from any of the 297 genomic DNA samples tested, the p*SSU* region was amplified from 29 (9.8%) of them. SSCP-based analysis of amplicons revealed three unique banding profiles. In total, nine amplicons representing these three profiles were sequenced. No sequence variation was detected among all three sequences representing each SSCP profile, such that, finally, three unique p*SSU* sequences (GenBank accession nos. KF891292-KF891294) were defined. These three new sequence types differed by 84-97% upon pairwise comparison. Based on the phylogenetic analysis, two sequence types (accession nos. KF891293 and KF891294) were genetically most similar but not identical to *Cryptosporidium* genotypes reported previously from water buffaloes (see Figure 1). The other sequence type defined was the same as the p*SSU* sequence of *Cryptosporidium* with accession no. KF891292 recorded in cattle in the present study (see section on *Cryptosporidium* species/genotypes in cattle). In the epidemiological context (Table 1), the *Cryptosporidium* sequence types with accession nos. KF891293, KF891294 and KF891292 were detected in 2.4% (farms BR, NK and MR), 5.7% (farms HA and MR) and 1.7% (farm MR) of the 297 samples tested, respectively.

*Cryptosporidium* was detected on all four water buffalo farms (Table 2). Samples test-positive for *Cryptosporidium* were detected in 25 (8.4%) and four (1.3%) of 297 samples from animals in the categories of < 6 months and ≥ 6 months of age, respectively (Table 3). The lowest (2.4%) and highest (17.6%) percentages of samples test-positive for *Cryptosporidium* were detected on farms NK and HA, respectively, but there was no significant difference in numbers of test-positive samples between the two different climatic regions in which samples were collected. The total number of calves (< 6 months) that were test-positive for *Cryptosporidium* was significantly lower for water buffaloes than for cattle in the study population (*P <* 0.001).

**Table 2:** ***Cryptosporidium *** **genotypes and *Giardia duodenalis *** **assemblage E detected in faecal samples from buffaloes from four farms in Sri Lanka**

**Farm**	**Total**	***Cryptosporidium *** genotypes*			***Giardia duodenalis *** assemblage E*
		**Genotype 9 (KF891292)**	**Genotype 10 (KF891293)**	**Genotype 11 (KF891294)**	
					**(KF891311 and KF891312)**
HA	51	0	0	9	1
BR	58	0	4	0	0
NK	82	0	2	0	0
MR	106	5	1	8	1
Total	297	5	7	17	2

*GenBank accession nos. are given within round parentheses.

**Table 3:** ***Cryptosporidium *** **genotypes and *Giardia duodenalis *** **assemblage E detected in faecal samples collected from two different age groups of water buffaloes in Sri Lanka**

**Age group**	**Total**	***Cryptosporidium *** genotypes*			***Giardia duodenalis *** assemblage E*
		**Genotype 9 (KF891292)**	**Genotype 10 (KF891293)**	**Genotype 11 (KF891294)**	
					**(KF891311 and KF891312)**
<6 months	108	5	5	15	1
≥6 months	189	0	2	2	1
Total	297	5	7	17	2

*GenBank accession nos. are given in round parentheses.

### Detection of *Giardia duodenalis* in cattle

**Figure 2:**
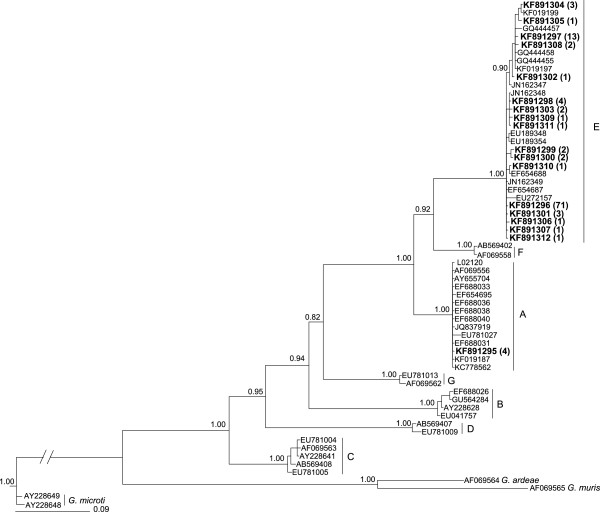
**Phylogenetic relationships of p*tpi *sequences of *Giardia duodenalis *based on Bayesian inference (BI) analysis.** Sixteen sequences determined in the present study, and 41 reference sequences representing *G. duodenalis* assemblages A to G (accession nos. listed in Additional file 4) were included in the analysis. Sequences representing *G. ardeae*, *G. muris* and *G. microti* were used as outgroups. Accession numbers linked to sequences determined in the present study are in bold-type; the numbers of samples with particular sequence types are in parentheses. Posterior probabilities (pp) are indicated at all major nodes.

All 340 faecal genomic DNA samples were subjected to genetic analysis for *Giardia*. The p*tpi* locus was amplified from 141 (41.5%) of these samples. SSCP analysis of all 141 amplicons displayed 16 distinct profiles. The direct sequencing of 60 amplicons representing all 16 profiles defined 16 distinct sequence types (GenBank accession nos. KF891295-KF891310). These 16 sequences differed by 86-99% upon pairwise comparison. All sequences (~530 bp) representing the 16 profiles were compared with publicly available sequences. One of the 16 sequences was identical to the reference sequence (accession number L02120) for *G. duodenalis* assemblage A. The 15 other sequences were identified as *G. duodenalis* assemblage E (Table 4). Three of the 15 sequences were identical to *G. duodenalis* assemblage E sequences with accession nos. JN162349, JN162348 and EF654688, respectively. Twelve other sequences were 99% similar to *G. duodenalis* assemblage E sequences with GenBank accession nos. JN162349 (n = 5), JN162348 (n = 2), JN162347 (n = 3) and GQ444455 (n = 2). A comprehensive phylogenetic analysis (Figure 2) supported their classification; the 15 and one sequences determined in this study clustered, with strong nodal support (pp = 1.00), with *G. duodenalis* assemblages E and A, respectively (Figure 2).

**Table 4:** **Fifteen different p** ***tpi *** **sequences representing assemblage E of *Giardia duodenalis *** **detected in cattle and buffaloes in Sri Lanka**

**Farm**	**Host**	Numbers of samples (accession nos.)*	**Subtotal**
AB	Cattle (*Bos taurus*)	47 (KF891296), 3 (KF8912967), 3 (KF891298), 2 (KF891299), 2 (KF891300), 2 (KF891301), 1 (KF891302)	60
NZ	Cattle	20 (KF891296), 10 (KF891297), 3 (KF891304), 2 (KF891303), 1 (KF891305), 1 (KF891306), 1 (KF891307), 1 (KF891298)	39
DY	Cattle	29 (KF891298), 4 (KF891296), 2 (KF891308), 1 (KF891301), 1 (KF891309), 1 (KF891310)	38
HA	Water buffalo (*Bubalus bubalis*)	1 (KF891311)	1
MR	Water buffalo	1 (KF891312)	1
BR	Water buffalo	0	0
NK	Water buffalo	0	0
		Total	139

*GenBank accession nos. are indicated in round parentheses.

These results were then put into an epidemiological context. *Giardia* was detected molecularly in 141 (22.1%) samples originating from all three cattle farms. Assemblages A and E were identified in four and 137 of these samples. The highest percentage of test-positive samples was detected in cattle on farm AB (51.3%), followed by farms DY (40.8%) and NZ (32.5%). Each cattle farm had 19 calves that were test-positive for *Giardia* at some stage during the sampling period. Mixed infections of *Cryptosporidium* and *Giardia* were detected in 98 (28.8%) of 340 samples. Of 60 cattle from all three farms, 57 had mixed infections of *Cryptosporidium* and *Giardia* at least once during the 6-week sampling period. The total number of calves (< 6 months) that were test-positive for *Giardia* was significantly higher for cattle than for water buffaloes (P < 0.001).

### Detection of *Giardia duodenalis* in water buffalo

All 297 faecal genomic DNA samples were subjected to genetic analysis for *Giardia*. The p*tpi* locus was amplified from two (0.7%) of these samples; the two samples were from water buffaloes of < 1 year of age (farms HA and MR: ≥6 months of age on both farms). The two amplicons were sequenced directly and compared with publicly available sequences. The sequences (GenBank accession nos. KF891311 and KF891312) were the same as two reference sequences (accession nos. JN162348 and JN162349) representing *G. duodenalis* assemblage E (Additional file 4). The two samples test-positive for *Giardia* also both contained a *Cryptosporidium* sequence type with accession no. KF891294.

## Discussion

This study genetically characterised, for the first time, *Cryptosporidium* and *Giardia* from *Bos taurus* and *Bubalus bubalis* in Sri Lanka. *Cryptosporidium* was identified in 62.1% and 9.8% of 340 samples, and *Giardia* in 41.5% and 0.7% of 279 samples from these two respective bovid hosts. Overall, two *Cryptosporidium* species (*C. bovis* and *C. ryanae*) and eight new genotypes were defined based on their p*SSU* sequence (n = 5, accession nos. KF891287-KF891291 in cattle; n = 2, KF891293 and KF891294 in water buffalo; and n = 1, KF891292 in both cattle and buffalo). *C. bovis* and *C. ryanae* were detected in calves of 10 and 19 days of age, respectively, time points which are relatively consistent with the pre-patent periods reported for these species of *Cryptosporidium*[47]. Although both *C. bovis* and *C. ryanae* (previously called *Cryptosporidium* “deer-like genotype”) have been widely reported in calves from different countries throughout the world [48,49], the eight genotypes characterised here are novel. Consistent with the findings for p*SSU*, no zoonotic species or genotypes of *Cryptosporidium* were detected using p*gp60*, in the absence of any evidence of PCR inhibition.

The first new *Cryptosporidium* genotype represented by accession no. KF891287, which is distinct in p*SSU* sequence by one nucleotide substitution (G - > C at alignment position 220; Additional file 3) from *C. ryanae* (accession no. EU410344), was most commonly detected (44.5%) amongst the 211 *Cryptosporidium* test-positive samples from cattle. This genotype was detected at least once on all three cattle farms studied and at least once during the 6-week sampling period. The second most frequent (28.9%), new *Cryptosporidium* genotype pertaining to accession no. KF891290 differed in sequence by one nucleotide substitution (G - > C at position 220; Additional file 3) from *C. bovis* (AY741305) and was also detected on all three cattle farms examined.

The third and fourth new *Cryptosporidium* genotypes (accession nos. KF891288 and KF891289), which both differed by two nucleotides (insertion of T or A at alignment positions 73 and 74, and a G - > C at position 220; Additional file 3) from *C. ryanae*, were each recorded in four and five samples, respectively, from farms AB and DY. These two genotypes differed by only one nucleotide (G- > C) from previously reported *C. ryanae* variants (accession nos. KC778534 and KC778535; [50]).

The fifth new genotype (accession no. KF891291), which differed in sequence by one nucleotide from *C. andersoni*, was identified in one sample. Phylogenetic analysis (Figure 1) revealed that the sequence representing this genotype formed a monophyletic group, with five reference sequences representing *C. andersoni* (pp = 0.88). Although *C. andersoni* occurs in adult cattle, it has been found occasionally in pre-weaned calves [51-55]. The sixth new genotype (accession no. KF891292) was identified in two faecal samples from one calf on one farm (NZ) and five samples isolated from water buffaloes from another farm (MR). This genotype is one nucleotide different from the p*SSU* sequence of a new *Cryptosporidium* genotype described previously in cattle in Australia (accession no. KC778530; [50]), Denmark (accession no. DQ182599; [56]), India (accession no. GQ345008; [57]) and the UK (accession no. HQ822134; [58]), and also in water buffaloes in Australia (accession no. KF019204; [29]). Published sequence data for the heat shock protein 70 (*hsp*70) and actin genes also supported the validity of this new genotype [58]. Therefore, this genotype might represent a new species, but it remains to be described.

The seventh and eighth new genotypes (represented by accession nos. KF891293 and KF891294) recorded from water buffaloes differed in sequence by a single point mutation (G- > C at position 220; Additional file 3) from genotypes 1 and 2 (accession nos. KF019202 and KF019203) described recently [29] in water buffaloes in Australia, and they were genetically most similar to those represented by accession nos. KF019202, KF019203, AB712388 and JQ002562 [29,59,60] in the phylogenetic analysis. These results indicate that these two novel genotypes of *Cryptosporidium* are buffalo-affiliated, but these genotypes are clearly different from *C. ryanae* from cattle [47].

Most samples (86.2% of 29) test-positive for *Cryptosporidium* in water buffaloes were detected in calves of <6 months of age. This result is consistent with previous studies [29,61,62], in which the occurrence of *Cryptosporidium* infection has been reported to be higher in water buffaloes of <6 months than in those of ≥6 months of age. A novel genotype represented by accession no. KF891294 was most frequently detected (58.6%) among *Cryptosporidium* test-positive samples from water buffaloes. It was also detected more frequently in samples collected from calves of <6 months of age group than those of ≥6 months age group. All of the eight novel genotypes identified here in cattle and buffaloes from Sri Lanka had a unique single-nucleotide (G < − > C) substitution in p*SSU* (Additional file 3) and appear to be autochthonous to this country.

Surprisingly, *C. parvum*, the most commonly reported zoonotic species of *Cryptosporidium* recognised in cattle throughout the world [2,15,48], was not detected in the present study in any of the samples collected from calves of 1 week to 3 months of age using a repeated sampling strategy (every week for six weeks). According to epidemiological studies conducted in many countries [16,52,55,63-67], *C. parvum* is most commonly detected in calves of <3 months of age. According to a longitudinal survey conducted by Santín and colleagues [55], *C. parvum* was detected in 85% of 503 pre-weaned calves, whereas only 1% was associated with post-weaned calves. For this reason, pre-weaned calves are recognised as the major reservoir for human cryptosporidiosis [2]. However, some recent epidemiological studies have reported an abundance of *C. bovis* infection and a limited presence of *C. parvum* in calves of 1–60 days of age in China [23,68,69], India [68] Nigeria [70] and Sweden [71]. Taken together, the present findings suggest that calves of this age group are not reservoirs for human *C. parvum* infection in the geographical regions studied here in Sri Lanka. A likely explanation for this unexpected result might relate to sound husbandry practices on farms in Sri Lanka. The three cattle farms studied were well-managed, intensive farms, and pre-weaned calves were kept and fed/watered in individual, elevated wooden calf pens with slatted floors.

*G. duodenalis* assemblage A was detected in only four samples from calves on one dairy farm (DY), whereas 97% of samples test-positive for *Giardia* in cattle related to *G. duodenalis* assemblage E. This result is consistent with the previous studies [50,72-76] reporting that 80-100% of the *G. duodenalis* isolated from cattle are in assemblage E. This assemblage was detected in calves on all three dairy farms and, at least once, in individual calves during the 6-week period of sampling. The two samples test-positive for *Giardia* in water buffaloes on farms HA and MR contained *G. duodenalis* assemblage E, although *Giardia* was not detected on the other two farms. Previous molecular studies conducted in Australia [29] and Italy [28] have also reported *G. duodenalis* assemblage E in water buffaloes. These findings support the proposal that cattle and water buffaloes in the geographical areas studied in Sri Lanka are not significant reservoirs for human infection with *Giardia*.

Both *Cryptosporidium* and *Giardia* were detected concurrently in 28.8% of 340 samples from cattle, which is in accordance with previous studies of cattle [77-79]. For each genus, the numbers of test-positive samples were higher in cattle than in water buffaloes, which seems to be consistent with a prevalence of up to 100% recorded in studies of dairy calves [22] compared with 9.5-38.3% in water buffalo [28,29,61,80-82].

## Conclusions

Results of the present study suggest that the epidemiology of bovine cryptosporidiosis and giardiasis in Sri Lanka is distinct from those of other parts of the world. Unique nucleotide substitutions in the p*SSU* region appear to be specific to Sri Lanka. Expanded studies of domestic and wild bovids in various regions in Sri Lanka are required to test this proposal. The apparent absence of *C. parvum* from cattle and buffaloes and the low occurrence of *G. duodenalis* assemblage A in cattle suggest that bovids in the regions studied here have limited significance as reservoirs for human infections. Future work should be focused on large-scale studies to investigate the molecular epidemiology of cryptosporidiosis and giardiasis in a wide range of animals in Sri Lanka and in neighbouring countries in South Asia.

## Competing interests

The authors declare that they have no competing interests.

## Authors’ contributions

HA carried out molecular laboratory work, data analysis, interpretation and drafting of the manuscript. KU collected samples. AVK assisted with the phylogenetic analyses. HA, RBG and ARJ wrote the manuscript with critical input from RPVJR and other authors. RBG and ARJ conceived the project and attracted the funding. All authors read and approved the final version of the manuscript.

## Supplementary Material

Additional file 1 **Summary of salient information (*Cryptosporidium *** **species/genotypes, host/environmental source, country, GenBank accession nos. of sequences and associated references) pertaining to the *SSU *** **sequences used in the phylogenetic analysis of p** ***SSU *** **data (see Figure **1**).** Click here for file

Additional file 2 **Pairwise comparison of nucleotide sequence differences in the small subunit of nuclear ribosomal RNA (p** ***SSU*) among *Cryptosporidium*** **species or genotypes representing reference sequences (GenBank accession nos. EU410344, AY741305, EU245042, EF489038, EU331243 and AB712384) and those from bovids studied herein (bold-type).** Click here for file

Additional file 3 **An alignment of known reference sequences representing a part of the small subunit of nuclear ribosomal RNA (p*SSU*) of *Cryptosporidium *species or genotypes (GenBank accession nos. EU410344, AY741305, EU245042, EF489038, EU331243 and AB712384) with homologous sequences derived from *Cryptosporidium *from bovids in the present study.** A dot denotes a nucleotide that is identical to that in the top sequence; a dash represents a gap. Click here for file

Additional file 4 **Summary of salient information (*Giardia* species/assemblages, host origins, country, accession nos. of sequences and associated references) pertaining to the *tpi *** **sequences used in the phylogenetic analysis of p** ***tpi * data (see Figure **2**).** Click here for file
