# Supplementary material for: First molecular characterization of Cryptosporidium and Giardia from bovines (Bos taurus and Bubalus bubalis) in Sri Lanka: unexpected absence of C. parvum from pre-weaned calves
Source: Parasit Vectors. 2014 Feb 21;7:75. doi: 10.1186/1756-3305-7-75 (PMC4015788; doi:10.1186/1756-3305-7-75)
Supplement: Additional file 1 — Summary of salient information ( Cryptosporidium species/genotypes, host/environmental source, country, GenBank accession nos. of sequences and associated references) pertaining to the SSU sequences used in the phylogenetic analysis of p SSU data (see Figure 1). [file 1756-3305-7-75-S1.doc]

**Additional file 1** Summary of salient information (*Cryptosporidium* species/genotypes, host/environmental source, country, GenBank accession nos. of sequences and associated references) pertaining to the *SSU* sequences used in the phylogenetic analysis of p*SSU* data (see Figure 1).

| Species/genotype | Host or environmental source | Country | GenBank accession nos. | References |
| --- | --- | --- | --- | --- |
| *C. andersoni* | Cattle (*Bos taurus*) | Poland | KC685372 | [1] |
| *C. andersoni* | Cattle | India | JQ002564 | [2] |
| *C. andersoni* | Cattle | India | HM627525 | [3] |
| *C. andersoni* | Cattle | USA | GU831568 | [4] |
| *C. andersoni* | Cattle | USA | AF093496 | [5] |
| *C. baileyi* | Chicken (*Gallus domesticus*) | USA | AF093495 | [5] |
| *C. bovis* | Cattle | Sri Lanka | KF891286 | Present study |
| *C. bovis* | Cattle | USA | AY741305 | [6] |
| *C. bovis* | Cattle | United Kingdom | HQ822138 | [7] |
| *C. bovis* | Cattle | Brazil | EF493331 | [8] |
| *C. canis* | Dog (*Canis familiaris*) | USA | AF112576 | [9] |
| *C. cuniculus* | Human (*Homo sapiens*) | United Kingdom | EU437413 | [10] |
| *C. fayeri* | Red kangaroo (*Macropus rufus*) | Australia | AF159112 | [11] |
| *C. felis* | Human | United Kingdom | AF323566 | [12] |
| *C. felis* | Human | United Kingdom | HQ149021 | [13] |
| *C. fragile* | Black-spined toad (*Duttaphrynus melanostictus*) | Malaysia | EU162751 | [14] |
| *C. galli* | Capercaille (*Tetrao urogallus*) | Czech Republic | AY168848 | [15] |
| *C. hominis* | Human | Slovenia | AJ849464 | [16] |
| *C. hominis* | Cattle | India | HM627527 | [3] |
| *C. macropodum* | Eeastern grey kangaroo (*Macropus giganteus*) | Australia | AY237630 | [17] |
| *C. meleagridis* | Turkey (*Meleagris gallopavo*) | USA | AF112574 | [9] |
| *C.* cf. *molnari* | Guppy (*Poecilia reticulata*) | Australia | AY524773 | [18] |
| *C. muris* | Rock hyrax (*Procavia capensis*) | USA | AF093498 | [5] |
| *C. parvum* | Cattle | USA | AF093490 | [5] |
| *C. parvum* | Human | United Kingdom | GU971623 | [19] |
| *C. ryanae* | Cattle | Sri Lanka | KF891285 | Present study |
| *C. ryanae* | Cattle | Japan | AB513679 | [20] |
| *C. ryanae* | Cattle | USA | EU410344 | [21] |
| *C. ryanae* | Cattle | China | FJ463193 | [22] |
| *C. ryanae* | Cattle (assumed to be *Bos taurus*) | India | GQ345007 | [23] |
| *C. serpentis* | Corn snake (*Elaphe guttata*) | USA | AF151376 | [24] |
| *C. serpentis* | Savannah monitor (*Varanus exanthematicus*) | USA | AF093500 | [5] |
| *C. suis* | Pig (*Sus scrofa*) | Ireland | EF489038 | [25] |
| *C. suis* | Pig | China | GU254172 | [26] |
| *C. suis* | Pig | China | GQ227705 | [26] |
| *C. suis* | Pig | China | JF710253 | [27] |
| *C. ubiquitum* | Goat (*Capra aegagrus hircus*) | USA | HM209372 | [28] |
| *C. ubiquitum* | Water | Canada | JQ178279 | [29] |
| *C. varanii* | Corn snake | Germany | EF502042 | [30] |
| *C. wrairi* | Guinea pig (*Cavia porcellus*) | USA | AF115378 | [9] |
| *C. xiaoi* | Sheep (*Ovis aries*) | USA | FJ896046 | [31] |
| *C. xiaoi* | Sheep | Norway | GQ337962 | [32] |
| *C. xiaoi* | Sheep | China | GU014552 | [33] |
| Genotype 1 | Water buffalo (*Bubalus bubalis*) | Australia | KF019202 | [34] |
| Genotype 2 | Water buffalo | Australia | KF019203 | [34] |
| Genotype 3 | Water buffalo | Australia | KF019204 | [34] |
| Genotype 4 | Cattle | Sri Lanka | KF891287 | Present study |
| Genotype 5 | Cattle | Sri Lanka | KF891290 | Present study |
| Genotype 6 | Cattle | Sri Lanka | KF891288 | Present study |
| Genotype 7 | Cattle | Sri Lanka | KF891289 | Present study |
| Genotype 8 | Cattle | Sri Lanka | KF891291 | Present study |
| Genotype 9 | Cattle | Sri Lanka | KF891292 | Present study |
| Genotype 9 | Buffalo | Sri Lanka | KF891292 | Present study |
| Genotype 10 | Buffalo | Sri Lanka | KF891293 | Present study |
| Genotype 11 | Buffalo | Sri Lanka | KF891294 | Present study |
| New genotype similar to *C. suis* | Human | United Kingdom | HQ822146 | [7] |
| New genotype similar to *C. suis* | Cattle | India | GQ345008 | [23] |
| New genotype similar to *C. suis* | Cattle | Denmark | DQ182599 | [35] |
| Beaver | Beaver (*Castor canadensis*) | USA | EF641022 | [36] |
| Bear | Black bear (*Ursus americanus*) | USA | AF247535 | [37] |
| Chipmunk | Eastern chipmunk (*Tamias striatus*) | USA | EF641026 | [36] |
| Coyote | Coyote (*Canis latrans*) | USA | DQ385545 | [38] |
| Goose I | Canada goose (*Branta canadensis*) | USA | AY120912 | [39] |
| Goose II | Canada goose | USA | EF641009 | [36] |
| Koala | Koala (*Phascolarctos cinereus*) | Australia | AF108860 | [40] |
| Lizard | Leopard gecko (*Eublepharis macularius*) | Czech Republic | AY120915 | [39] |
| Mink | Mink (*Mustela vison*) | USA | EF641015 | [36] |
| Oppossum I | Oppossum (*Didelphis virginiana*) | USA | AY120902 | [39] |
| Ostrich | Ostrich (*Struthio camelus*) | Brazil | DQ002931 | [41] |
| Seal I | Ringed seal (*Phoca hispida*) | Canada | AY731234 | [42] |
| Shrew | Northern short-tailed shrew (*Blarina brevicauda*) | USA | EF641010 | [36] |
| Skunk | Human | United Kingdom | EU437415 | [10] |
| Snake | New Guinea boa (*Candoia aspera*) | USA | AY120913 | [39] |
| Tortoise | Star tortoise (*Geochelone* sp.) | USA | AY120914 | [39] |
| Wildebeest | Black wildebeest (*Connochaetes gnou*) | Portugal | AY883022 | [43] |
| *Eimeria tenella* | Chicken | USA | U67121 | [44] |
| *Eimeria necatrix* | Chicken | USA | U67119 | [44] |
| *Eimeria acervulina* | Chicken | USA | U67115 | [44] |
| *Eimeria brunetti* | Chicken | USA | U67116 | [44] |
|  |  |  |  |  |
