# Supplementary material for: First molecular characterization of Cryptosporidium and Giardia from bovines (Bos taurus and Bubalus bubalis) in Sri Lanka: unexpected absence of C. parvum from pre-weaned calves
Source: Parasit Vectors. 2014 Feb 21;7:75. doi: 10.1186/1756-3305-7-75 (PMC4015788; doi:10.1186/1756-3305-7-75)
Supplement: Additional file 2 — Pairwise comparison of nucleotide sequence differences in the small subunit of nuclear ribosomal RNA (p SSU ) among Cryptosporidium species or genotypes representing reference sequences (GenBank accession nos. EU410344, AY741305, EU245042, EF489038, EU331243 and AB712384) and those from bovids studied herein (bold-type). [file 1756-3305-7-75-S2.doc]

| *Cryptosporidium* species/ genotypes | EU410344  *C. ryanae* | AY741305  *C. bovis* | EU245042  *C. andersoni* | EF489038  *C. suis* | EU331243  Pig genotype II | AB712384  *C. parvum* | **KF891285**  ***C. ryanae*** | **KF891286**  ***C. bovis*** | **KF891287**  **genotype 4** | **KF891290**  **genotype 5** | **KF891288**  **genotype 6** | **KF891289**  **genotype 7** | **KF891291**  **genotype 8** | **KF891292**  **genotype 9** | **KF891293 genotype 10** |
| --- | --- | --- | --- | --- | --- | --- | --- | --- | --- | --- | --- | --- | --- | --- | --- |
| EU410344 *C. ryanae* |  |  |  |  |  |  |  |  |  |  |  |  |  |  |  |
| AY741305 *C. bovis* | 97 (3) |  |  |  |  |  |  |  |  |  |  |  |  |  |  |
| EU245042 *C. andersoni* | 84 (16) | 84 (16) |  |  |  |  |  |  |  |  |  |  |  |  |  |
| EF489038 *C. suis* | 89 (11) | 89 (11) | 83 (17) |  |  |  |  |  |  |  |  |  |  |  |  |
| EU331243 Pig genotype II | 93 (7) | 94 (6) | 82 (18) | 88 (12) |  |  |  |  |  |  |  |  |  |  |  |
| AB712384 *C. parvum* | 90 (10) | 90 (10) | 83 (17) | 95 (5) | 88 (12) |  |  |  |  |  |  |  |  |  |  |
| **KF891285 *C. ryanae*** | 100 (0) | 97 (3) | 84 (16) | 89 (11) | 93 (7) | 90 (10) |  |  |  |  |  |  |  |  |  |
| **KF891286 *C. bovis*** | 97 (3) | 100 (0) | 84 (16) | 89 (11) | 94 (6) | 90 (10) | 97 (3) |  |  |  |  |  |  |  |  |
| **KF891287 genotype 4** | 99 (1) | 96 (4) | 83 (17) | 89 (11) | 92 (8) | 89 (11) | 99 (1) | 96 (4) |  |  |  |  |  |  |  |
| **KF891290 genotype 5** | 96 (4) | 99 (1) | 83 (17) | 89 (11) | 93 (7) | 90 (10) | 96 (4) | 99 (1) | 97 (3) |  |  |  |  |  |  |
| **KF891288 genotype 6** | 99 (1) | 96 (4) | 84 (16) | 90 (10) | 92 (8) | 89 (11) | 99 (1) | 96 (4) | 100 (0) | 97 (3) |  |  |  |  |  |
| **KF891289 genotype 7** | 99 (1) | 96 (4) | 83 (17) | 89 (11) | 92 (8) | 89 (11) | 99 (1) | 96 (4) | 100 (0) | 97 (3) | 99 (1) |  |  |  |  |
| **KF891291 genotype 8** | 84 (16) | 84 (16) | 99 (1) | 84 (16) | 80 (20) | 81 (19) | 84 (16) | 84 (16) | 84 (16) | 84 (16) | 85 (15) | 84 (16) |  |  |  |
| **KF891292 genotype 9** | 89 (11) | 89 (11) | 81 (19) | 99 (1) | 87 (13) | 96 (4) | 89 (11) | 89 (11) | 89 (11) | 89 (11) | 89 (11) | 90 (10) | 82 (18) |  |  |
| **KF891293 genotype 10** | 99 (1) | 97 (3) | 83 (17) | 89 (11) | 93 (7) | 89 (11) | 99 (1) | 97 (3) | 99 (1) | 97 (3) | 98 (2) | 99 (1) | 84 (16) | 89 (11) |  |
| **KF891294 genotype 11** | 99 (1) | 97 (3) | 84 (16) | 88 (12) | 93 (7) | 90 (10) | 99 (1) | 97 (3) | 99 (1) | 97 (3) | 99 (1) | 99 (1) | 85 (15) | 89 (11) | 99 (1) |

**Additional file 3** Pairwise comparison of nucleotide sequence differences in the small subunit of nuclear ribosomal RNA (p*SSU*) (~220 bp) among *Cryptosporidium* species or genotypes representing reference sequences (GenBank accession nos. EU410344, AY741305, EU245042, EF489038, EU331243 and AB712384) and those from bovids studied herein (bold-type).
