# Supplementary material for: First molecular characterization of Cryptosporidium and Giardia from bovines (Bos taurus and Bubalus bubalis) in Sri Lanka: unexpected absence of C. parvum from pre-weaned calves
Source: Parasit Vectors. 2014 Feb 21;7:75. doi: 10.1186/1756-3305-7-75 (PMC4015788; doi:10.1186/1756-3305-7-75)
Supplement: Additional file 3 — An alignment of known reference sequences representing a part of the small subunit of nuclear ribosomal RNA (pSSU) of Cryptosporidium species or genotypes (GenBank accession nos. EU410344, AY741305, EU245042, EF489038, EU331243 and AB712384) with homologous sequences derived from Cryptosporidium from bovids in the present study. A dot denotes a nucleotide that is identical to that in the top sequence; a dash represents a gap. [file 1756-3305-7-75-S3.doc]

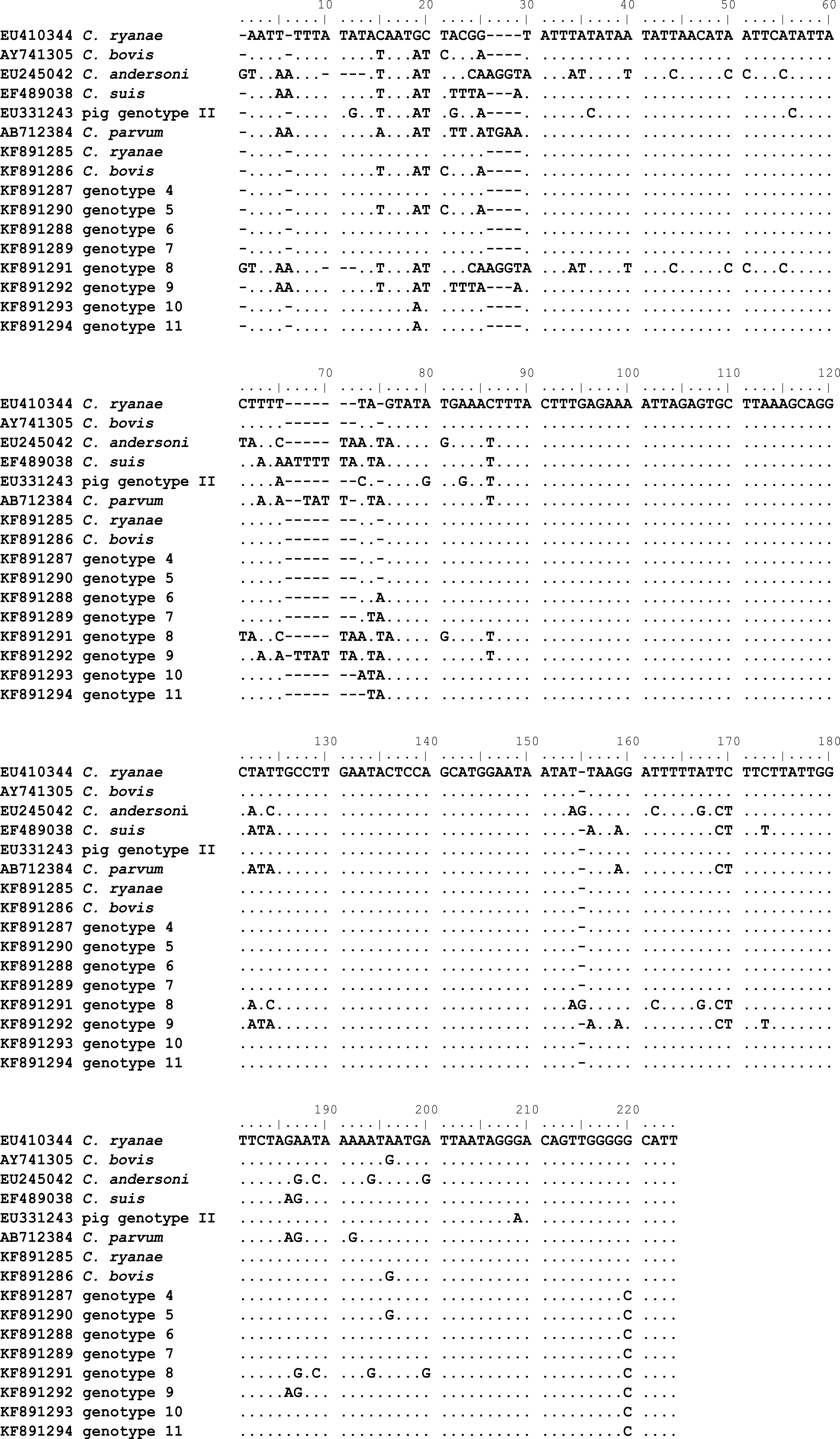


**Additional file 4** – Abeywardena *et al*.

**Legend to Additional file 4:**

An alignment of known reference sequences representing a part of the small subunit of nuclear ribosomal RNA (p*SSU*) of *Cryptosporidium* species or genotypes (GenBank accession nos. EU410344, AY741305, EU245042, EF489038, EU331243 and AB712384) with homologous sequences derived from *Cryptosporidium* from bovids in the present study. A dot denotes a nucleotide that is identical to that in the top sequence; a dash represents a gap.
