# Supplementary material for: First molecular characterization of Cryptosporidium and Giardia from bovines (Bos taurus and Bubalus bubalis) in Sri Lanka: unexpected absence of C. parvum from pre-weaned calves
Source: Parasit Vectors. 2014 Feb 21;7:75. doi: 10.1186/1756-3305-7-75 (PMC4015788; doi:10.1186/1756-3305-7-75)
Supplement: Additional file 4 — Summary of salient information ( Giardia species/assemblages, host origins, country, accession nos. of sequences and associated references) pertaining to the tpi sequences used in the phylogenetic analysis of p tpi data (see Figure 2). [file 1756-3305-7-75-S4.doc]

**Additional file 4** Summary of salient information (*Giardia* species/assemblages, host origins, country, accession nos. of sequences and associated references) pertaining to the *tpi* sequences used in the phylogenetic analysis of p*tpi* data (see Figure 2).

| Species/assemblage | Host origin | Country | Accession numbers | References |
| --- | --- | --- | --- | --- |
| *G. duodenalis* |  |  |  |  |
| A | Cattle (*Bos taurus*) | Sri Lanka | KF891295 | Present study |
|  | Human (*Homo sapiens*) | Afghanistan | L02120 | [45] *cf.* [46] |
|  | Human | Australia | AF069556 | [47] *cf.* [48] |
|  | Human | Australia | EF688031 | [49] |
|  | Human | Canada | EF688033 | [49] |
|  | Human | Egypt | EF688036 | [49] |
|  | Human | Israel | EF688040 | [49] |
|  | Human | Peru | EF688038 | [49] |
|  | Cattle | Australia | KC778562 | [50] |
|  | Cattle | New Zealand | JQ837919 | [51] |
|  | Cattle | USA | AY655704 | [52] |
|  | Cattle | USA | EF654695 | [53] |
|  | Buffalo (*Bubalus bubalis*) | Australia | KF019187 | [34] |
|  | Cat (*Felis catus*) | Sweden | EU781027 | [54] |
| B | Human | Algeria | EU041757 | [55] |
|  | Human | Australia | EF688026 | [49] |
|  | Human | China | GU564284 | [56] |
|  | Human | Peru | AY228628 | [57] |
| C | Dog (*Canis familiaris*) | Australia | AF069563 | [47] *cf.* [58] |
|  | Dog | Japan | AB569408 | [59] |
|  | Dog | Sweden | EU781005, EU781004 | [54] |
|  | Dog | USA | AY228641 | [57] |
| D | Dog | Japan | AB569407 | [59] |
|  | Dog | Sweden | EU781009 | [54] |
| E | Cattle | Sri Lanka | KF891296-KF891310 | Present study |
|  | Buffalo | Sri Lanka | KF891311, KF891312 | Present study |
|  | Human | Egypt | EU272157 | [60] |
| F | Cat | Australia | AF069558 | [47] *cf.* [61] |
|  | Cat | Japan | AB569402 | [59] |
| G | Rat (*Rattus norvegicus*) | Australia | AF069562 | [47] |
|  | Rat | Sweden | EU781013 | [54] |
| *G. muris* | Mouse (*Mus musculus*) | USA | AF069565 | [47] *cf.* [62] |
| *G. ardeae* | Blue Heron (*Ardea herodias*) | USA | AF069564 | [47] *cf.* [63] |
| *G. microti* | Muskrat (*Ondatra zibethicus*) | USA | AY228648, AY228649 | [57] |
|  |  |  |  |  |
